# Occurrence of intraocular hemorrhages under monotherapy or combination therapy of antiplatelets and anticoagulants using the Japanese Adverse Drug Event Report database

**DOI:** 10.3389/jpps.2023.11263

**Published:** 2023-04-12

**Authors:** Junko Tanaka, Takenao Koseki, Kohsuke Sekido, Masashi Kimata, Yasuki Ito, Shigeki Yamada

**Affiliations:** ^1^ Department of Pharmacotherapeutics and Informatics, Fujita Health University School of Medicine, Toyoake, Aichi, Japan; ^2^ Department of Ophthalmology, Fujita Health University School of Medicine, Toyoake, Aichi, Japan

**Keywords:** intraocular hemorrhages, antiplatelets, anticoagulants, monotherapy, combination therapy

## Abstract

**Purpose:** An intraocular hemorrhage is an adverse event that can lead to visual acuity impairment. Antithrombotic therapy with antiplatelet agents and anticoagulants may increase intraocular hemorrhage. However, since their frequency is low, studies on the risk of intraocular hemorrhage with these drugs, especially under combination therapy, are limited. This study aimed to investigate the occurrence of intraocular hemorrhages under monotherapy and combination therapy with antiplatelets and anticoagulants by analyzing a large pharmacovigilance database.

**Methods:** Intraocular hemorrhage signals with oral antiplatelets and anticoagulants were evaluated by calculating reporting odds ratios and information components using the Japan Adverse Drug Reactions Report database from April 2004 to March 2022. In addition, differences in signals between younger and elderly patients, affecting factors, and time-to-onset from initial antiplatelet and anticoagulant treatments were analyzed.

**Results:** Aspirin, clopidogrel, warfarin, apixaban, and rivaroxaban, but not ticagrelor, ticlopidine, prasugrel, dabigatran, and edoxaban showed intraocular hemorrhage signals under monotherapy. In combination therapy, dual therapy (aspirin + P2Y_12_ inhibitors, warfarin, direct oral anticoagulants, and P2Y_12_ inhibitors + warfarin) and triple therapy (aspirin + P2Y_12_ inhibitors + warfarin) resulted in intraocular hemorrhage signals. Intraocular hemorrhage signals were observed in younger patients receiving monotherapy with aspirin and in elderly patients receiving monotherapy and combination therapy with warfarin. Affecting factors were diabetes mellitus in patients with prasugrel, use of medications for intravitreal injections, and posterior sub-Tenon injections with some antiplatelets and anticoagulants. The median period of intraocular hemorrhage occurrence after starting monotherapy with aspirin, clopidogrel, warfarin, or rivaroxaban was within 90 days.

**Conclusion:** In addition to monotherapy with several antiplatelets and anticoagulants, combination therapy using aspirin, P2Y_12_ inhibitors, and warfarin has the potential risk of intraocular hemorrhage. Particular attention should be paid to the occurrence of intraocular hemorrhages in younger patients taking aspirin, in elderly patients taking warfarin, and within the first 90 days of antiplatelet and anticoagulant use.

## Introduction

The use of antiplatelets (APs), such as aspirin and P2Y_12_ inhibitors, and anticoagulants (ACs), such as warfarin and Direct Oral Anticoagulants (DOACs), is increasing as the elderly grow to prevent recurrent thrombotic and ischemic events in patients with coronary artery disease (1,2). While it is well known that APs and ACs increase the bleeding risk, including severe, life-threatening bleeding, intraocular hemorrhages (IOHs) are also bleeding risks that require attention because, although their occurrence is rare, they can cause visual acuity impairment and decrease the patient’s quality of life (3–6). For example, a meta-analysis including randomized clinical trials for atrial fibrillation or venous thromboembolism reported that apixaban showed a trend with an increased IOHs event rate compared to warfarin (5). However, another meta-analysis reported that the risk of IOHs did not differ between warfarin and other DOACs in patients with atrial fibrillation included in randomized clinical trials (3). Thus, in addition to the different reports in similar meta-analyses, these reports require further investigation because of the small number of cases of IOHs. In addition, few reports have investigated the risk of IOHs associated with APs and ACs, distinguishing between monotherapy and combination therapy. Combination therapy with aspirin and P2Y_12_ inhibitors (dual antiplatelet therapy; DAPT) is widely implemented as a first-line treatment strategy in patients with acute coronary syndromes and those undergoing percutaneous coronary intervention. Additionally, APs and ACs are often used in combination therapy and monotherapy (7). Analyzing the risk of IOHs under monotherapy and combination therapy with APs and ACs would allow for a more comprehensive of APs and ACs risk management given their clinical use.

In recent years, pharmacovigilance approaches for drug-associated adverse event (AE) signal detection using large databases, such as the Japanese Adverse Drug Event Report (JADER) based on spontaneous AE reports, have evolved (8). Drug-associated AE signals are evaluated through disproportionality analyses of frequency statistics, such as calculating the reporting odds ratios (RORs) and information components (ICs) of pharmacovigilance activity (8,9). JADER is a nationwide open-access database of spontaneous reports of AE provided by the Pharmaceuticals and Medical Devices Agency (PMDA), a pharmaceutical regulatory authority in Japan. As the JADER database contains approximately 780,000 patients and 1,280,000 AEs reported after April 2004, it is a useful tool for detecting signals of rare AEs, such as IOHs, in patients receiving APs and ACs.

It has been reported that warfarin and some DOACs under monotherapy showed signals of choroidal hemorrhage, retinal hemorrhage, and vitreous hemorrhage using Vigibase, a pharmacovigilance database obtained from the World Health Organization (4). However, it is unclear that the IOHs signaling is observed in patients treated with APs and ACs in combination therapy.

In this study, we investigated the signals of IOHs during monotherapy or combination therapy using JADER database. In addition, we investigated differences in IOHs signals in terms of age, affecting factors for occurrence of IOHs, and time to onset of IOHs in patients taking oral APs and ACs.

## Materials and methods

### Data source

**FIGURE 1:**
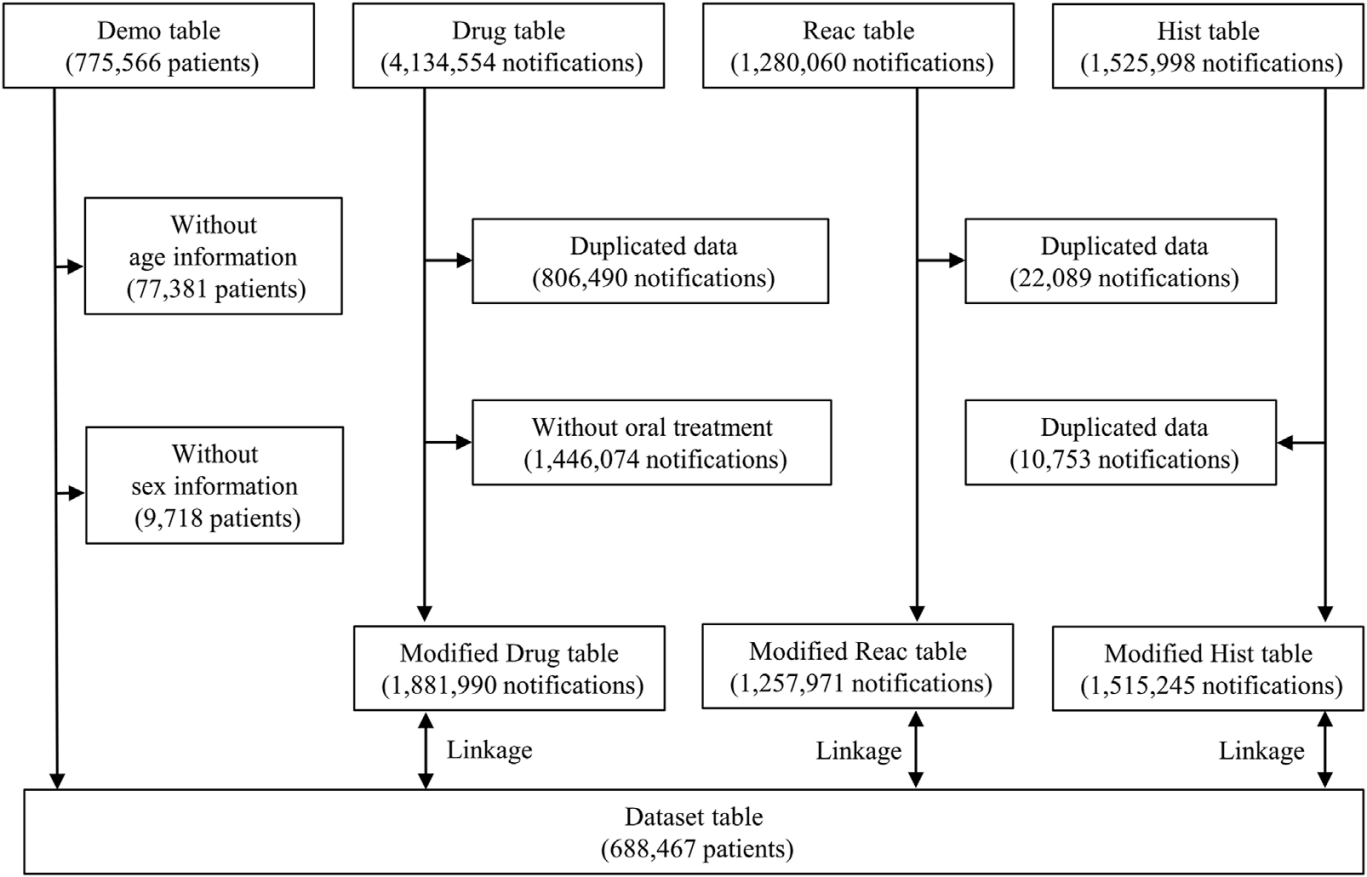
Flow diagram of the study.

Data between April 2004 and March 2022 from the JADER database were obtained from the PMDA website^1^. The JADER dataset comprised four data tables: the demographic information “demo” table, drug information “drug” table, AE information “reac” table, and primary disease information “hist” table, which included 775,566 patients, 4,134,554 notifications, 1,280,060 notifications, and 1,525,998 notifications. The “demo” table included the sex and age information of patients. Patients with blanked/unknown sex or age data in the “demo” table and notifications with duplicated data in the “drug,” “reac,” and “hist” tables were excluded. In the “drug” table, the contribution of the drugs to the AEs was classified into three categories: suspected drug, concomitant drug, and interaction. In this study, all categories included the influence of APs and ACs as concomitant drugs and interactions. Those administered *via* the “oral” route were selected to evaluate the signals for IOHs in patients who received APs and ACs orally. The “demo” table was linked to the “drug,” “reac,” and “hist” tables using the patient identification number of each case. After data cleaning, 688,467 patients were included in this study (Figure 1).

For subgroup analysis, younger patients were defined as those in the “under 10s,” “10s,” “20s,” “30s,” “40s,” “50s,” and “60s” age groups and elderly patients were defined as those in the “70s,” “80s,” “90s,” and “100s” age groups, as previously reported (9).

### Targeted antiplatelets and anticoagulants

The study included ten APs and ACs approved for use in Japan: cyclooxygenase inhibitor; Aspirin, P2Y_12_ inhibitors; clopidogrel sulfate, ticagrelor, ticlopidine hydrochloride, prasugrel hydrochloride, vitamin K antagonist; Warfarin potassium, DOACs; apixaban, dabigatran etexilate methanesulfonate, edoxaban tosylate hydrate, and rivaroxaban. The route of administration was selected to be “oral.”

### Definition of intraocular hemorrhages

IOHs were extracted from the “reac” table according to the preferred terms (PTs) in the Medical Dictionary for Regulatory Activities (MedDRA, 25.0 J). Seventeen PTs were determined as IOHs by two ophthalmologists from PTs grouped into the high level term (HLT) and Ocular haemorrhagic disorders (HLT code 10064464). IOHs because of external or physical reasons were excluded (Table 1).

**TABLE 1:** Definition of intraocular hemorrhages.

HLT code	HLT name
10064464	Ocular haemorrhagic disorders
PT code	PT name
10008786	Choroidal haemorrhage
10010719	Conjunctival haemorrhage
10015926	Eye haemorrhage
10020923	Hyphaema
10030919	Optic disc haemorrhage
10038867	Retinal haemorrhage
10047655	Vitreous haemorrhage
10050508	Scleral haemorrhage
10051558	Corneal bleeding
10057417	Ciliary body haemorrhage
10057418	Iris haemorrhage
10068642	Choroidal haematoma
10071934	Intraocular haematoma
10071935	Subretinal haematoma
10071936	Vitreous haematoma
10079891	Eye haematoma
10085163	Scleral haematoma

HLT, high level term; PT, preferred term.

### Signal detection

This study used ROR and IC to evaluate the signal detection of IOHs, as previously reported (9,10). The ROR is an AE signal index, the odds ratio of reporting a particular AE versus all other AEs associated with the target drugs, compared to the reporting odds for all other drugs. However, the results obtained using RORs might be unreliable when the sample size is small. IC is an ADR signal index of the Bayesian Confidence Propagation Neural Network analysis, calculated based on the Bayesian statistical approach. Therefore, it can detect AE signals when the sample size is small (8,9,11). RORs, ICs, and their 95% confidence intervals (CIs) were calculated using a two-by-two contingency table (Table 2) and the equations described below.

**TABLE 2:** Two-by-two contingency table.

	Target AEs	Other AEs	Total
Target drugs	*N*_*11*_	*N*_*10*_	*N*_*1+*_
Other drugs	*N*_*01*_	*N*_*00*_	*N*_*0+*_
Total	*N*_*+1*_	*N*_*+0*_	*N*_*++*_

AEs, adverse events; *N*, number of patients.

ROR equations:

$$ROR=\frac{N_{11}/N_{01}}{N_{10}/N_{00}}=\frac{N_{11}N_{00}}{N_{10}N_{01}}$$

$$ROR\left(95\%\;CI\right)=e^{\ln\left(ROR\right)\pm 1.96\sqrt{\frac{1}{N_{11}}+\frac{1}{N_{10}}+\frac{1}{N_{01}}+\frac{1}{N_{00}}}}$$

IC Equations:

$$E\left({IC}_{11}\right)=\log_{2}\frac{\left(N_{11}+\gamma_{11}\right)\left(N_{++}+\alpha \right)\left(N_{++}+\beta \right)}{\left(N_{++}+\gamma \right)\left(N_{+1}+\alpha_{1}\right)\left(N_{+1}+\beta_{1}\right)}$$

$$V\left({IC}_{11}\right)={\left(\frac{1}{\ln2}\right)}^{2}[\frac{N_{++}-N_{11}+\gamma -\gamma_{11}}{\left(N_{11}+\gamma_{11}\right)\left(1+N_{++}+\gamma \right)}+\frac{N_{++}-N_{+1}+\alpha -\alpha_{1}}{\left(N_{1+}+\alpha_{1}\right)\left(1+N_{++}+\alpha \right)}+\frac{N_{++}-N_{+1}+\beta -\beta_{1}}{\left(N_{+1}+\beta_{1}\right)\left(1+N_{++}+\beta \right)}]$$

$$\gamma =\gamma_{11}\frac{\left(N_{++}+\alpha \right)\left(N_{++}+\beta \right)}{\left(N_{+1}+\alpha_{1}\right)\left(N_{+1}+\beta_{1}\right)}\gamma_{11}=1,\alpha_{1}=\beta_{1}=1,\alpha =\beta =2$$

$$IC\left(95\%\;CI\right)=E\left({IC}_{11}\right)\pm 2\sqrt{V\left({IC}_{11}\right)}$$

The calculations were performed using Microsoft 365 (Microsoft Corporation). The signals for IOHs were positive when the lower limit of the 95% CI of the ROR exceeded 1, and that of the IC exceeded 0.

### Factor analysis

To investigate factors affecting the occurrence of IOHs, multivariable logistic regression analysis was performed in patients reported for AP and AC use, as in a previous report (12). Predictive factors examined included the comorbidity of hypertension, diabetes mellitus, and reported use of medications for intravitreal injections (IVI) and posterior sub-Tenon triamcinolone acetonide injections (PSTAI). Hypertension and diabetes mellitus as primary diseases were extracted from the “hist” table according to PTs in the MedDRA, 25.0 J. Each PT was determined from Hypertension (code 20000147) and Hyperglycaemia/new onset diabetes mellitus (code 20000041) in the Standardized MedDRA Queries (SMQ) (Tables 3, 4). Medications for IVI and PSTAI were selected from those approved in Japan: aflibercept, brolucizumab, faricimab, ranibizumab, and triamcinolone acetonide. For triamcinolone acetonide, the route of administration was selected as “intraocular injections,” “intravitreal injections” and “posterior sub-Tenon injections.” *p* values <0.05 were considered statistically significant. Statistical analysis was performed using EZR (Saitama Medical Center, Jichi Medical University, Saitama, Japan) as a graphical user interface for R version 4.1.2 (The R Foundation for Statistical Computing, Vienna, Austria). EZR is a modified version of R Commander (version 1.55, designed to add statistical functions frequently used in biostatistics (13).

**TABLE 3:** Definition of hypertension.

SMQ code	SMQ name
20000147	Hypertension
PT code	PT name
10000358	Accelerated hypertension
10005732	Blood pressure ambulatory increased
10005739	Blood pressure diastolic increased
10051128	Blood pressure inadequately controlled
10005750	Blood pressure increased
10063926	Blood pressure management
10053355	Blood pressure orthostatic increased
10005760	Blood pressure systolic increased
10081751	Catecholamine crisis
10063067	Dialysis induced hypertension
10012758	Diastolic hypertension
10014129	Eclampsia
10057615	Endocrine hypertension
10015488	Essential hypertension
10070538	Gestational hypertension
10049058	HELLP syndrome
10020571	Hyperaldosteronism
10020772	Hypertension
10049781	Hypertension neonatal
10059238	Hypertensive angiopathy
10020801	Hypertensive cardiomegaly
10058222	Hypertensive cardiomyopathy
10077000	Hypertensive cerebrovascular disease
10020802	Hypertensive crisis
10058179	Hypertensive emergency
10020803	Hypertensive encephalopathy
10079496	Hypertensive end-organ damage
10020823	Hypertensive heart disease
10055171	Hypertensive nephropathy
10058181	Hypertensive urgency
10049079	Labile hypertension
10025600	Malignant hypertension
10025603	Malignant hypertensive heart disease
10026674	Malignant renal hypertension
10026924	Maternal hypertension affecting foetus
10026985	Mean arterial pressure increased
10052066	Metabolic syndrome
10067598	Neurogenic hypertension
10065508	Orthostatic hypertension
10076704	Page kidney
10050631	Postoperative hypertension
10036485	Pre-eclampsia
10065918	Prehypertension
10062886	Procedural hypertension
10038464	Renal hypertension
10074864	Renal sympathetic nerve ablation
10038562	Renovascular hypertension
10038926	Retinopathy hypertensive
10039808	Secondary aldosteronism
10039834	Secondary hypertension
10084825	Superimposed pre-eclampsia
10078932	Supine hypertension
10042957	Systolic hypertension
10048007	Withdrawal hypertension

PT, preferred term; SMQ, Standardized MedDRA Queries.

**TABLE 4:** Definition of diabetes mellitus.

SMQ code	SMQ name
20000041	Hyperglycaemia/new onset diabetes mellitus
PT code	PT name
10087376	Acquired generalised lipodystrophy
10065367	Blood 1,5-anhydroglucitol decreased
10005557	Blood glucose increased
10012596	Diabetes complicating pregnancy
10012601	Diabetes mellitus
10012607	Diabetes mellitus inadequate control
10012631	Diabetes with hyperosmolarity
10077357	Diabetic arteritis
10012650	Diabetic coma
10080788	Diabetic coronary microangiopathy
10071265	Diabetic hepatopathy
10012668	Diabetic hyperglycaemic coma
10012669	Diabetic hyperosmolar coma
10012671	Diabetic ketoacidosis
10012672	Diabetic ketoacidotic hyperglycaemic coma
10012673	Diabetic ketosis
10074309	Diabetic metabolic decompensation
10081558	Diabetic wound
10080061	Euglycaemic diabetic ketoacidosis
10017395	Fructosamine increased
10072628	Fulminant type 1 diabetes mellitus
10018209	Gestational diabetes
10018429	Glucose tolerance impaired
10018430	Glucose tolerance impaired in pregnancy
10018478	Glucose urine present
10082836	Glycated albumin increased
10087214	Glycated serum protein increased
10018473	Glycosuria
10018475	Glycosuria during pregnancy
10018481	Glycosylated haemoglobin abnormal
10018484	Glycosylated haemoglobin increased
10085610	Hepatogenous diabetes
10020635	Hyperglycaemia
10087319	Hyperglycaemic crisis
10063554	Hyperglycaemic hyperosmolar nonketotic syndrome
10071394	Hyperglycaemic seizure
10071286	Hyperglycaemic unconsciousness
10056997	Impaired fasting glucose
10022489	Insulin resistance
10022491	Insulin resistant diabetes
10053247	Insulin-requiring type 2 diabetes mellitus
10023379	Ketoacidosis
10023388	Ketonuria
10023391	Ketosis
10023392	Ketosis-prone diabetes mellitus
10066389	Latent autoimmune diabetes in adults
10086189	Maternally inherited diabetes and deafness
10075980	Monogenic diabetes
10028933	Neonatal diabetes mellitus
10086425	Neonatal hyperglycaemia
10082630	New onset diabetes after transplantation
10033660	Pancreatogenous diabetes
10081755	Steroid diabetes
10067584	Type 1 diabetes mellitus
10067585	Type 2 diabetes mellitus
10072659	Type 3 diabetes mellitus
10057597	Urine ketone body present

PT, preferred term; SMQ, Standardized MedDRA Queries.

### Time-to-onset analysis

Time-to-onset analysis was performed using the periods from the initial administration of APs and ACs in the “drug” table to the date of the first occurrence of the IOHs recorded in the “reac” table. Patients with missing or inaccurate data regarding the initial administration of APs and ACs and the date of the first occurrence of IOHs were excluded. When the period of IOHs occurrence exceeded 720 d, the calculation was performed for 720 d. The median period and interquartile range (IQR) were calculated using JMP 13.0 (SAS Institute Inc., Cary, NC, USA). We evaluated APs and ACs in >10 patients reporting IOHs between the day of the initial dose and the day of the first occurrence of IOHs.

## Results

### Intraocular hemorrhages signal under monotherapy

The RORs and ICs of APs and ACs for IOHs under monotherapy are shown in Table 5. Except for ticagrelor, one or more IOHs were reported in the APs and ACs. Among APs, aspirin (ROR, 1.40 [95% CI 1.08 to 1.80] and IC, 0.45 [95% CI 0.08 to 0.83]) and clopidogrel (ROR, 3.20 [95% CI 2.32 to 4.43] and IC, 1.57 [95% CI 1.10 to 2.04]) showed IOHs signals. In ACs, warfarin (1.60 [95% CI 1.17 to 2.19] and IC, 0.64 [95% CI 0.19 to 1.10]), apixaban (ROR, 4.79 [95% CI 3.73 to 6.16] and IC, 2.12 [95% CI 1.76 to 2.49]), and rivaroxaban (ROR, 8.84 [95% CI 7.21 to 10.85] and IC, 2.94 [95% CI 2.64 to 3.24] showed IOHs signals.

**TABLE 5:** Reporting odds ratios and information components of each antiplatelets and anticoagulants under monotherapy for intraocular hemorrhages.

	Total AEs	IOHs	ROR	95% CI	IC	95% CI
Antiplatelets						
Aspirin	19,200	61	**1.40**	**1.08 to 1.80**	**0.45**	**0.08 to 0.83**
P2Y_12_ inhibitors						
Clopidogrel	5,260	38	**3.20**	**2.32 to 4.43**	**1.57**	**1.10 to 2.04**
Ticagrelor	39	0	NA		−0.13	−3.05 to 2.79
Ticlopidine	2,018	8	1.73	0.86 to 3.47	0.67	−0.30 to 1.64
Prasugrel	166	2	5.29	1.31 to 21.34	1.12	−0.57 to 2.80
Anticoagulants						
Warfarin	11,229	41	**1.60**	**1.17 to 2.19**	**0.64**	**0.19 to 1.10**
DOACs						
Apixaban	6,040	64	**4.79**	**3.73 to 6.16**	**2.12**	**1.76 to 2.49**
Dabigatran	2,864	10	1.52	0.82 to 2.84	0.53	−0.34 to 1.41
Edoxaban	3,255	10	1.34	0.72 to 2.49	0.37	−0.50 to 1.25
Rivaroxaban	5,288	100	**8.84**	**7.21 to 10.85**	**2.94**	**2.64 to 3.24**

Bold indicates RORs and ICs for drugs that showed intraocular hemorrhage signal. AEs, adverse events; CI, confidence interval; DOACs, direct oral anticoagulants; IC, information component; IOHs, intraocular hemorrhages; NA, not applicable; ROR, reporting odds ratio.

### Intraocular hemorrhages signal under combination therapy

The RORs and ICs of APs and ACs in combination therapy are shown in Table 6. In combination therapy with two types of antithrombotic agents (dual therapy), aspirin showed IOHs signals with all types of antithrombotic agents (with P2Y_12_ inhibitors: ROR, 3.55 [95%CI 2.74 to 4.60] and IC, 1.72 [95% CI 1.34 to 2.10]; with warfarin: ROR, 3.12 [95% CI 2.10 to 4.63] and IC, 1.51 [95% CI 0.93 to 2.08]; with DOACs: ROR, 3.64 [95% CI 2.15 to 6.18] and IC, 1.61 [95% CI 0.86 to 2.37]). P2Y_12_ inhibitors showed IOHs signals in combination with warfarin (ROR, 3.96 [95% CI 2.24 to 7.00] and IC, 1.67 [95% CI 0.87 to 2.48]), but not DOACs (ROR, 2.29 [95% CI 1.02 to 5.11] and IC, 0.94 [95% CI −0.15 to 2.04]). In combination therapy with three types of antithrombotic agents (triple therapy): DAPT with warfarin or DOACs, an IOHs signal was detected in combination with warfarin (DAPT with warfarin: ROR, 6.16 [95% CI 3.29 to 11.52] and IC, 2.05 [95% CI 1.17 to 2.93]), but not with DOACs (ROR, 2.09 [95% CI 0.52 to 8.39] and IC, 0.61 [95% CI −1.06 to 2.28]).

**TABLE 6:** Reporting odds ratios and information components of antiplatelets and anticoagulants under combination therapy for intraocular hemorrhages.

		Total AEs	IOHs	ROR	95% CI	IC	95% CI
Aspirin	+P2Y_12_ inhibitors	7,582	60	**3.55**	**2.74 to 4.60**	**1.72**	**1.34 to 2.10**
	+Warfarin	3,538	25	**3.12**	**2.10 to 4.63**	**1.51**	**0.93 to 2.08**
	+DOACs	1,690	14	**3.64**	**2.15 to 6.18**	**1.61**	**0.86 to 2.37**
P2Y_12_ inhibitors	+Warfarin	1,333	12	**3.96**	**2.24 to 7.00**	**1.67**	**0.87 to 2.48**
	+DOACs	1,144	6	2.29	1.02 to 5.11	0.94	−0.15 to 2.04
Aspirin + P2Y_12_ inhibitors	+Warfarin	717	10	**6.16**	**3.29 to 11.52**	**2.05**	**1.17 to 2.93**
	+DOACs	417	2	2.09	0.52 to 8.39	0.61	−1.06 to 2.28

Bold indicates RORs and ICs for drugs that showed intraocular hemorrhage signal. ACs, anticoagulants; AEs, adverse events; APs, antiplatelets; CI, confidence interval; DOACs, direct oral anticoagulants; IC, information component; IOHs, intraocular hemorrhages; ROR, reporting odds ratio.

### Difference in intraocular hemorrhage signal with age

The RORs and ICs of APs and ACs that showed IOHs signals under monotherapy or combination therapy in the younger/elderly groups are shown in Table 7. In monotherapy, aspirin showed an IOHs signal in the younger group (ROR, 2.14 [95% CI 1.47 to 3.10], IC, 1.01 [95% CI 0.47 to 1.55]). Warfarin showed IOHs signal in the elderly group (ROR, 1.52 [95% CI 1.04 to 2.22] and IC, 0.56 [95% CI 0.01 to 1.11]). In dual therapy, aspirin with warfarin (ROR, 3.51 [95% CI 2.25 to 5.48] and IC, 1.62 [95% CI 0.97 to 2.26]), DOACs (ROR, 3.11 [95% CI 1.75 to 5.51] and IC, 1.40 [95% CI 0.59 to 2.21]), and P2Y_12_ inhibitors with warfarin (ROR, 4.16 [95% 2.22 to 7.79] and IC, 1.67 [95% CI 0.79 to 2.55]) showed IOHs signals in the elderly group. In the combination therapy of DAPT with warfarin, the IOHs signal was detected in the elderly group (ROR, 8.20 [95% CI 4.37 to 15.41] and IC, 2.28 [95% CI 1.40 to 3.17]).

**TABLE 7:** Reporting odds ratios and information components of antiplatelets and anticoagulants under monotherapy or combination therapy for intraocular hemorrhages in age-based subgroup.

		Age	Total AEs	IOHs	ROR	95%CI	IC	95%CI
APs	Aspirin	**Younger**	**7,178**	**29**	**2.14**	**1.47 to 3.10**	**1.01**	**0.47 to 1.55**
		Elderly	12,022	32	0.93	0.66 to 1.33	−0.09	−0.60 to 0.42
	Clopidogrel	**Younger**	**1,547**	**16**	**5.49**	**3.34 to 9.03**	**2.09**	**1.38 to 2.80**
		**Elderly**	**3,713**	**22**	**2.12**	**1.39 to 3.25**	**0.99**	**0.38 to 1.61**
ACs	Apixaban	**Younger**	**1,116**	**12**	**5.69**	**3.21 to 10.09**	**2.04**	**1.23 to 2.85**
		**Elderly**	**4,924**	**52**	**3.94**	**2.97 to 5.23**	**1.82**	**1.41 to 2.23**
	Rivaroxaban	**Younger**	**1,241**	**34**	**15.17**	**10.71 to 21.49**	**3.36**	**2.86 to 3.87**
		**Elderly**	**4,047**	**66**	**6.26**	**4.85 to 8.06**	**2.42**	**2.05 to 2.79**
	Warfarin	Younger	4,659	13	1.45	0.84 to 2.52	0.48	−0.30 to 1.26
		**Elderly**	**6,570**	**28**	**1.52**	**1.04 to 2.22**	**0.56**	**0.01 to 1.11**
Aspirin	+P2Y_12_ inhibitors	**Younger**	**2,913**	**30**	**5.55**	**3.85 to 8.01**	**2.22**	**1.69 to 2.76**
		**Elderly**	**4,669**	**30**	**2.32**	**1.61 to 3.35**	**1.12**	**0.59 to 1.65**
	+Warfarin	Younger	1,479	5	1.76	0.73 to 4.24	0.64	−0.55 to 1.82
		**Elderly**	**2,059**	**20**	**3.51**	**2.25 to 5.48**	**1.62**	**0.97 to 2.26**
	+DOACs	Younger	308	2	3.38	0.84 to 13.62	0.91	−0.77 to 2.59
		**Elderly**	**1,382**	**12**	**3.11**	**1.75 to 5.51**	**1.40**	**0.59 to 2.21**
P2Y_12_ inhibitors	+Warfarin	Younger	470	2	2.21	0.55 to 8.88	0.65	−1.02 to 2.33
		**Elderly**	**863**	**10**	**4.16**	**2.22 to 7.79**	**1.67**	**0.79 to 2.55**
Aspirin + P2Y_12_ inhibitors	+Warfarin	Younger	274	0	NA		−0.62	−3.51 to 2.28
		**Elderly**	**443**	**10**	**8.20**	**4.37 to 15.41**	**2.28**	**1.40 to 3.17**

Bold indicates RORs and ICs for drugs that showed intraocular hemorrhage signal. AEs, adverse events; ACs, anticoagulants; APs, antiplatelets; CI, confidence interval; DOACs, direct oral anticoagulants; IC, information component; IOHs, intraocular hemorrhages; NA, not applicable; ROR, reporting odds ratio.

### Factors affecting intraocular hemorrhages

To investigate factors affecting the occurrence of IOHs under monotherapy or combination therapy of APs and ACs, we assessed the effect of comorbidity of hypertension, diabetes mellitus, and use of medications for IVI and PSTAI on the occurrence of IOHs (Table 8). Multivariable analysis revealed that hypertension and diabetes mellitus were not detected as affecting factors for IOHs in almost all of APs and ACs, excluding prasugrel (diabetes mellitus; OR, 6.37 [95% CI 1.71–23.70], *p* = 0.006). IVI and PSTAI increased the frequency of IOHs in most APs and ACs with their presence.

**TABLE 8:** Multivariable analysis for affecting factors of intraocular hemorrhages with antiplatelets and anticoagulants.

	Multivariable analysis		
	OR	95% CI	*p*-values
Aspirin			
Hypertension	0.76	0.52–1.09	0.138
Diabetes mellitus	1.19	0.80–1.76	0.388
IVI + PSTAI	36.7	19.40–69.40	<0.001
Clopidogrel			
Hypertension	0.71	0.41–1.22	0.212
Diabetes mellitus	0.71	0.38–1.33	0.280
IVI + PSTAI	9.26	1.20–71.4	0.033
Ticlopidine			
Hypertension	0.50	0.14–1.78	0.284
Diabetes mellitus	0.25	0.03–1.93	0.182
IVI + PSTAI	NA		
Prasugrel			
Hypertension	2.33	0.63–8.69	0.207
Diabetes mellitus	6.37	1.71–23.70	0.006
IVI + PSTAI	64.7	3.10–1350.00	0.007
Warfarin			
Hypertension	1.15	0.67–1.97	0.609
Diabetes mellitus	0.58	0.26–1.30	0.185
IVI + PSTAI	36.2	12.20–108.00	<0.001
Apixaban			
Hypertension	1.19	0.70–2.04	0.517
Diabetes mellitus	0.40	0.14–1.14	0.087
IVI + PSTAI	NA		
Dabigatran			
Hypertension	1.82	0.61–5.46	0.287
Diabetes mellitus	1.72	0.52–5.76	0.376
IVI + PSTAI	NA		
Edoxaban			
Hypertension	1.37	0.42–4.46	0.604
Diabetes mellitus	1.51	0.38–5.90	0.558
IVI + PSTAI	NA		
Rivaroxaban			
Hypertension	0.69	0.45–1.04	0.078
Diabetes mellitus	1.37	0.82–2.28	0.225
IVI + PSTAI	NA		

CI, confidence intervals; IVI, intravitreal injections; NA, not applicable; OR, odds ratio; PSTAI, posterior sub-Tenon triamcinolone acetonide injections.

### Time-to-onset analysis

In the time-to-onset analysis, two APs (aspirin and clopidogrel) monotherapy and three ACs (warfarin potassium, apixaban, rivaroxaban) monotherapy with >10 reports of IOH were included in the evaluation. The median period [IQR] to the onset of IOHs in patients with APs and ACs was as follows: aspirin (72.5 [30.3–369.5] days), clopidogrel (79.5 [12.3–443.8] days), warfarin (29.0 [14.0–367.0] days), apixaban (174.0 [34.0–381.5] days), and rivaroxaban (90.0 [21.8–339.5] days). The time-to-onset of IOHs for combination therapy could not be evaluated because no more than 10 patients reported IOHs between initial administration and the date of the first occurrence of IOHs (Table 9).

**TABLE 9:** Time to intraocular hemorrhages occurrence and case distribution of occurrence period under monotherapy of antiplatelets and anticoagulants.

		Median	IQR	Cases	≤D2	D3-7	D8-30	D31-60	D61-90	D91-180	D181-360	D361-
APs	Aspirin	72.5	30.3–369.5	10	0	1	1	2	2	1	1	2
	Clopidogrel	79.5	12.3–443.8	15	0	3	1	2	2	2	0	5
ACs	Warfarin	29.0	14.0–367.0	10	0	1	2	0	1	0	0	6
	Apixaban	174.0	34.0–381.5	24	0	3	2	1	1	4	4	9
	Rivaroxaban	90.0	21.8–339.5	52	5	2	8	5	7	6	7	12

ACs, anticoagulants; APs, antiplatelets; CI, confidence intervals; D, day; IQR, Interquartile range.

## Discussion

The signals of IOHs under monotherapy and combination therapy by APs and ACs, signal differences in ages, affecting factors, and their time-to-onset were analyzed using the JADER pharmacovigilance database. IOHs signals were shown in both monotherapies with aspirin and combination therapy of aspirin with P2Y_12_ inhibitors, warfarin, and DOACs. It has been reported that monotherapy of aspirin, and combination therapy taking more than 1 aspirin/clopidogrel/warfarin are associated with an increased risk of the IOHs occurrence in patients with neovascular age-related macular degeneration who had no previous intraretinal hemorrhage and no history of trauma, posterior segment surgery (14). Sun et al. also reported in a meta-analysis that aspirin and P2Y_12_ inhibitors (prasugrel or ticagrelor) did not increase the risk of IOHs compared to aspirin and other P2Y_12_ inhibitor (clopidogrel). However, their study did not assess whether DAPT increases the risk of IOHs (15). In addition to these reports, our results show that IOHs may be more likely to occur in patients receiving aspirin monotherapy and combination therapy with aspirin and P2Y_12_ inhibitors (DAPT), warfarin, or DOACs, alerting us to the risk of IOHs during aspirin monotherapy and combination therapy. In P2Y_12_ inhibitors monotherapy, clopidogrel but not other P2Y_12_ inhibitors, such as prasugrel, showed IOHs signals. In contrast, a retrospective study reported that the prevalence of spontaneous IOHs on prasugrel was higher than that on clopidogrel (16). In our study, prasugrel showed a high ROR, but no IOHs signal. This result may be because of the low number of cases of prasugrel in all AEs and the lack of signal detection, which needs to be reevaluated after case accumulation. In APs monotherapy, warfarin, apixaban, and rivaroxaban showed IOHs signals. These results are consistent with previous reports that warfarin increases spontaneous IOHs compared to age-matched controls (17), apixaban showed a signal of retinal hemorrhage based on Vigibase (4); rivaroxaban showed a higher prevalence of spontaneous IOH than dabigatran, clopidogrel, and ticagrelor (16). Contrarily, Sun et al. reported in a meta-analysis of integrated analysis of DOACs (apixaban, dabigatran, edoxaban, or rivaroxaban) that DOACs reduce the risk of IOH compared to warfarin (18). Their meta-analysis was an integrated analysis of randomized clinical trials that compared warfarin and did not assess the risk of IOH for the DOACs themselves. Therefore, according to our study, attention should be paid to the risk of potential IOHs in patients taking apixaban, rivaroxaban, or warfarin. Similar to DAPT, IOHs signals were observed in combination therapy P2Y_12_ inhibitors with warfarin, but not P2Y_12_ inhibitors and DOACs. In addition, triple combination therapy of DAPT with warfarin, but not DAPT with DOACs, resulted in IOHs signals. In combination therapy of DAPT with DOACs, all AEs were reported more frequently with apixaban among DOACs, but these did not include IOHs (data not shown), a trend that differed from that observed with apixaban monotherapy. In fact, the combination therapy of apixaban with P2Y_12_ inhibitors was associated with a lower risk of thrombolysis in myocardial infarction major and minor bleeding, trial-defined primary bleeding events, and intracranial hemorrhage compared with the combination therapy of P2Y_12_ inhibitors with warfarin in meta-analysis (19), and the risk of IOHs with combination therapy of apixaban requires further investigation. Overall, the results of this study show that both monotherapy and combination therapy with APs and ACs increase the potential risk of IOHs.

In the age-based subgroup analysis, IOHs signals were observed in elderly patients receiving warfarin monotherapy and combination therapy. These results are consistent with reports that spontaneous IOHs occurrence was higher in elderly patients receiving warfarin treatment (17) and combination therapy of warfarin with APs (20). In contrast, IOHs signals were observed in younger patients receiving aspirin monotherapy but not in elderly patients. A meta-analysis examining the safety of aspirin in the primary prevention of cardiovascular disease found no significant differences in major bleeding, intracranial hemorrhage, or gastrointestinal bleeding between younger and elderly patients (21). Furthermore, in a prospective population-based cohort study of patients with a transient ischemic attack, ischemic stroke, or myocardial infarction treated with APs (mainly aspirin-based), the long-term risk of major bleeding was higher in elderly patients than in younger patients (22). Although these reports show that the risk of bleeding with aspirin is greater in the elderly, the risk of IOHs has not been evaluated and should be investigated. Our results showed that even in younger patients, aspirin use should be done with caution regarding the potential risk of IOHs.

Hypertension, diabetes mellitus, IVI, and PSTAI are known to affect the occurrence of IOHs (23–25). In our study, diabetes mellitus increased the frequency of IOHs in prasugrel, but no other APs and ACs affected the comorbidity of hypertension and diabetes mellitus. IVI and PSTAI also increased the frequency of IOHs, as expected. Thus, attention should be paid to the potential risk of IOHs during AP and AC therapy, regardless of the presence or absence of hypertension or diabetes mellitus.

In the time-to-onset analysis, the median period of IOHs occurrence after starting monotherapy with APs and ACs, excluding apixaban, was within 90 days, and the 75-percentile period of IOHs occurrence with APs and ACs was approximately 360 days. The recommended period of DAPT after coronary artery diseases, such as acute coronary syndromes and percutaneous coronary intervention, is 1–12 months, depending on the pathophysiology, thrombotic risk, and bleeding risk of the target patients, followed by monotherapy with APs or ACs (1,26). We have not been able to analyze the onset time of IOHs under combination therapy such as DAPT because of the limited number of evaluable IOHs. However, based on the monotherapy estimation, the occurrence of IOH should be noted during the first year after starting APs or ACs therapy, especially during the first 90 days.

Our study had several limitations. First, spontaneous reporting systems, such as JADER, are passive reporting systems, and there are many biases, including under-reporting, over-reporting, and confounding by comorbidities. In addition to hypertension, diabetes mellitus, IVI, and PSTAI, intraocular surgery such as vitrectomy is considered a risk factor for IOHs. Because the JADER database does not include information on the presence of surgical history, the influence of intraocular surgery cannot be ruled out. Second, the influence of AP and ACs doses cannot be ruled out. For example, aspirin exhibits antiplatelet effects when administered at low doses; however, the limited information on drug doses in JADER makes accurate assessment difficult. Third, the number of cases of ticagrelor and prasugrel use and the number of evaluable cases in the time-to-onset analysis was small. Finally, we defined an IOHs signal as one with a significant difference in RORs and ICs to avoid false positive detection. Future investigations with more case-accumulated databases are needed to assess the IOHs signal and time-to-onset.

## Conclusion

In conclusion, the potential risk of IOHs increases during monotherapy with aspirin, clopidogrel, warfarin, apixaban, and rivaroxaban and during combination therapy with most APs and ACs, such as DAPT. In addition, aspirin use in younger patients, warfarin use in the elderly, and within the first 90 days of APs and ACs use should be considered for the occurrence of IOHs. Our results may help internists, ophthalmologists, pharmacists, and other medical staff predict and manage the occurrence of IOH under APs and ACs monotherapy and combination therapy.

## Data Availability

Publicly available datasets were analyzed in this study. This data can be found here: https://www.pmda.go.jp/safety/info-services/drugs/adr-info/suspected-adr/0003.html.

## Footnotes

1
https://www.pmda.go.jp/english/index.html

## Abbreviations

AC, anticoagulant; AE, adverse event; AP, antiplatelet; CI, confidence interval; DOAC, Direct Oral Anticoagulant; HLT, high level term; IC, information component; IOH, intraocular hemorrhage; IQR, interquartile range; IVI, intravitreal injections; JADER, Japanese Adverse Drug Event Report; PMDA, Pharmaceuticals and Medical Devices Agency; PSTAI, posterior sub-Tenon triamcinolone acetonide injections; PT, preferred term; ROR, reporting odds ratio; SMQ, Standardized MedDRA Queries.

## References

[1] Rodriguez F Harrington RA. Management of antithrombotic therapy after acute coronary syndromes. N Engl J Med (2021) 384:452–60. 10.1056/NEJMra1607714 33534976 PMC9275395

[2] Matsuura Y Moribayashi K Kaikita K. Optimal antithrombotic therapy in patients undergoing percutaneous coronary intervention: A focused review on high bleeding risk. J Atheroscler Thromb (2022) 29:1409–20. 10.5551/jat.RV17066 35934784 PMC9529379

[3] Caldeira D Barra M Pinto FJ Ferreira JJ Costa J. Intracranial hemorrhage risk with the new oral anticoagulants: A systematic review and meta-analysis. J Neurol (2015) 262:516–22. 10.1007/s00415-014-7462-0 25119841

[4] Talany G Guo M Etminan M. Risk of intraocular hemorrhage with new oral anticoagulants. Eye (Lond) (2017) 31:628–31. 10.1038/eye.2016.265 28009346 PMC5395993

[5] Phan K Lloyd D Wilson-Smith A Leung V Andric M. Intraocular bleeding in patients managed with novel oral anticoagulation and traditional anticoagulation: A network meta-analysis and systematic review. Br J Ophthalmol (2018) 103:641–7. 10.1136/bjophthalmol-2018-312198 29925514

[6] Garcia GA Bair H Kossler AL. Perioperative management of antithrombotic medications: An investigation into current U. S. Ophthalmologic recommendations. J Curr Ophthalmol (2021) 33:182–8. 10.4103/2452-2325.303201 34409230 PMC8365588

[7] Tantry US Navarese EP Myat A Chaudhary R Gurbel PA. Combination oral antithrombotic therapy for the treatment of myocardial infarction: Recent developments. Expert Opin Pharmacother (2018) 19:653–65. 10.1080/14656566.2018.1457649 29611444

[8] Nomura K Takahashi K Hinomura Y Kawaguchi G Matsushita Y Marui H Effect of database profile variation on drug safety assessment: An analysis of spontaneous adverse event reports of Japanese cases. Drug Des Dev Ther (2015) 9:3031–41. 10.2147/dddt.S81998 PMC4472069 26109846

[9] Tanaka J Koseki T Kondo M Ito Y Yamada S. Analyses of ocular adverse Reactions associated with anticancer drugs based on the Japanese pharmacovigilance database. Anticancer Res (2022) 42:4439–51. 10.21873/anticanres.15944 36039456

[10] Tanaka H Ohyama K Horikomi Y Ishii T. Association between anaphylaxis and anti-influenza drug use: An analysis of the Japanese Adverse Drug Event Report database. Drug Discoveries Ther (2021) 15:150–5. 10.5582/ddt.2021.01053 34234064

[11] Noguchi Y Murayama A Esaki H Sugioka M Koyama A Tachi T Angioedema caused by drugs that prevent the degradation of vasoactive peptides: A pharmacovigilance database study. J Clin Med (2021) 10:5507. 10.3390/jcm10235507 34884209 PMC8658484

[12] Koseki T Horie M Kumazawa S Nakabayashi T Yamada S. A pharmacovigilance approach for assessing the occurrence of suicide-related events induced by antiepileptic drugs using the Japanese adverse drug event report database. Front Psychiatry (2022) 13:1091386. 10.3389/fpsyt.2022.1091386 36699485 PMC9868764

[13] Kanda Y. Investigation of the freely available easy-to-use software 'EZR' for medical statistics. Bone Marrow Transpl (2013) 48:452–8. 10.1038/bmt.2012.244 PMC3590441 23208313

[14] Kiernan DF Hariprasad SM Rusu IM Mehta SV Mieler WF Jager RD. Epidemiology of the association between anticoagulants and intraocular hemorrhage in patients with neovascular age-related macular degeneration. Retina (2010) 30:1573–8. 10.1097/IAE.0b013e3181e2266d 21060269

[15] Sun MT Huang S Wiviott SD Antman EM Roe MT Ohman EM Meta-analysis of intraocular bleeding with dual antiplatelet therapy using P2Y12 inhibitors prasugrel or ticagrelor. Am J Cardiol (2020) 125:1280–3. 10.1016/j.amjcard.2020.01.012 32081368

[16] Sudarshana DM Konstantinou EK Arepalli S Silva FQ Schachat AP Ehlers JP The prevalence of adverse ocular hemorrhagic events in patients utilizing oral anticoagulant and antiplatelet therapy in routine clinical practice. Ophthalmic Surg Lasers Imaging Retina (2018) 49:27–34. 10.3928/23258160-20171215-04 29304263

[17] Biyik I Mercan I Ergene O Oto O. Ocular bleeding related to warfarin anticoagulation in patients with mechanical heart valve and atrial fibrillation. J Int Med Res (2007) 35:143–9. 10.1177/147323000703500116 17408066

[18] Sun MT Wood MK Chan W Selva D Sanders P Casson RJ Risk of intraocular bleeding with novel oral anticoagulants compared with warfarin: A systematic review and meta-analysis. JAMA Ophthalmol (2017) 135:864–70. 10.1001/jamaophthalmol.2017.2199 28687831 PMC5710315

[19] Liang B Zhu YC Gu N. Comparative safety and efficacy of eight antithrombotic regimens for patients with atrial fibrillation undergoing percutaneous coronary intervention. Front Cardiovasc Med (2022) 9:832164. 10.3389/fcvm.2022.832164 35387437 PMC8978794

[20] Kim KE Yang PS Jang E Kim S Joung B. Antithrombotic medication and the risk of vitreous hemorrhage in atrial fibrillation: Korean national Health insurance service national cohort. Yonsei Med J (2019) 60:65–72. 10.3349/ymj.2019.60.1.65 30554492 PMC6298896

[21] Calderone D Greco A Ingala S Agnello F Franchina G Scalia L Efficacy and safety of aspirin for primary cardiovascular risk prevention in younger and older age: An updated systematic review and meta-analysis of 173,810 subjects from 21 randomized studies. Thromb Haemost (2022) 122:445–55. 10.1055/a-1667-7427 34638150

[22] Li L Geraghty OC Mehta Z Rothwell PM. Age-specific risks, severity, time course, and outcome of bleeding on long-term antiplatelet treatment after vascular events: A population-based cohort study. Lancet (2017) 390:490–9. 10.1016/s0140-6736(17)30770-5 28622955 PMC5537194

[23] Jiang H Gao Y Fu W Xu H. Risk factors and treatments of suprachoroidal hemorrhage. Biomed Res Int (2022) 2022:1–5. 10.1155/2022/6539917 PMC9303137 35872859

[24] Tarlan B Kiratli H. Subconjunctival hemorrhage: Risk factors and potential indicators. Clin Ophthalmol (2013) 7:1163–70. 10.2147/opth.S35062 23843690 PMC3702240

[25] Ying GS Maguire MG Daniel E Grunwald JE Ahmed O Martin DF. Association between antiplatelet or anticoagulant drugs and retinal or subretinal hemorrhage in the comparison of age-related macular degeneration treatments trials. Ophthalmology (2016) 123:352–60. 10.1016/j.ophtha.2015.09.046 26545320 PMC4724480

[26] Levine GN Bates ER Bittl JA Brindis RG Fihn SD Fleisher LA 2016 ACC/AHA guideline focused update on duration of dual antiplatelet therapy in patients with coronary artery disease: A report of the American college of cardiology/American heart association task force on clinical practice guidelines: An update of the 2011 ACCF/AHA/SCAI guideline for percutaneous coronary intervention, 2011 ACCF/AHA guideline for coronary artery bypass graft surgery, 2012 ACC/AHA/ACP/AATS/PCNA/SCAI/STS guideline for the diagnosis and management of patients with stable ischemic heart disease, 2013 ACCF/AHA guideline for the management of ST-elevation myocardial infarction, 2014 AHA/ACC guideline for the management of patients with non–ST-elevation acute coronary syndromes, and 2014 ACC/AHA guideline on perioperative cardiovascular evaluation and management of patients undergoing noncardiac surgery. Circulation (2016) 134:e123–e155. 10.1161/cir.0000000000000404 27026020

